# CCNA2 as an Immunological Biomarker Encompassing Tumor Microenvironment and Therapeutic Response in Multiple Cancer Types

**DOI:** 10.1155/2022/5910575

**Published:** 2022-03-31

**Authors:** Aimin Jiang, Ye Zhou, Wenliang Gong, Xin Pan, Xinxin Gan, Zhenjie Wu, Bing Liu, Le Qu, Linhui Wang

**Affiliations:** ^1^Department of Urology, Changhai Hospital, Naval Medical University (Second Military Medical University), Shanghai, China; ^2^Department of Urology, Affiliated Jinling Hospital, Medical School of Nanjing University, Nanjing, Jiangsu Province, China; ^3^Department of Urology, The Third Affiliated Hospital of Second Military Medical University, Shanghai, China

## Abstract

**Background:**

Cancer is a major threat to human health worldwide. Although recent innovations and advances in early detection and effective therapies such as targeted drugs and immune checkpoint inhibitors have saved more lives of cancer patients and improved their quality of life, our knowledge about cancer remains largely unknown. CCNA2 belongs to the cell cyclin family and has been demonstrated to be a tumorigenic gene in multiple solid tumor types. The aim of the present study was to make a comprehensive analysis on the role of CCNA2 at a pancancer level.

**Methods:**

Multidatabases were collected to evaluate the different expression, prognostic value, DNA methylation, tumor mutation burden, microsatellite instability, mismatch repair, tumor immune microenvironment, and drug sensitivity of CCNA2 across pancancer. IHC was utilized to validate the expression and prognostic value of CCNA2 in ccRCC patients from SMMU cohort.

**Results:**

CCNA2 was differentially expressed in most cancer types vs. normal tissues. CCNA2 may significantly influence the prognosis of multiple cancer types, especially clear cell renal cell carcinoma (ccRCC). CCNA2 was also frequently mutated in most cancer types. Notably, CCNA2 was significantly correlated with immune cell infiltration and immune checkpoint inhibitory genes. In addition, CCNA2 was also strongly related to drug resistance.

**Conclusion:**

CCNA2 may prove to be a new biomarker for prognostic prediction, tumor immunity assessment, and drug susceptibility evaluation in pancancer level, especially in ccRCC.

## 1. Introduction

Cancer is one of the major diseases threatening human health [1]. With the continuous development of medical technology, the screening and treatment of cancer have improved constantly and remarkably [2]. However, only a few malignant tumor types could be completely cured at present due to the concealment and complexity of cancer occurrence and progression [3]. The application of targeted drugs has offered relatively good oncological outcomes in some tumors, but a considerable number of patients have innate drug resistance (DR) or develop DR after receiving treatment for a certain period [4, 5]. In recent years, immunotherapy has become an important means of cancer treatment. Immune checkpoint inhibitors bring hope to more patients [6, 7]. In the face of heterogeneity of cancer, target and immune therapy have limitation to control the progression of cancers once resistance or metastasis happens, leaving patients with few therapies to employ [8, 9]. Therefore, it is urgent to find new effective therapeutic targets.

CCNA2 (Cell cyclin A2) is located on the human chromosome 4, Q27 region, with a full length of 7489 bp, which is expressed in almost all tissues in the human body [10]. Generally, the protein coded by CCNA2 can activate cyclin-dependent kinase 2 (CDK2), thus promoting transition through G1/S and G2/M [11]; however, some studies have reported that CCNA2 might participate in the occurrence and progression of multiple tumors via affecting epithelial-mesenchymal transformation (EMT) and metastasis [12]. Recently, several studies have identified that CCNA2 might enhance cancer aggressive behavior, relapse, metastasis, and chemoresistance [13]. However, there is still no pancancer level analysis of the role of CCNA2 in various cancers, and the exact role of CCNA2 in tumorigenesis remains incompletely understood.

The aim of the present study was to explore the potential role of CCNA2 in tumor proliferation and analyze its correlation with the immune microenvironment in pancancer settings, especially in clear cell renal cell carcinoma (ccRCC) by conducting a comprehensive analysis of CCNA2 using several publicly available databases and inhouse datasets via bioinformatics and experiments.

## 2. Materials and Methods

### 2.1. Public Dataset Collection

The Cancer Genome Atlas (*TCGA*) database(http://cancergenome.nih.gov) is a comprehensive dataset containing multiple omics data of various cancers [14, 15]. TCGA database was utilized to download expression profiles, clinical information, mutation data, tumor mutation burden (TMB), and microsatellite instability (MSI) data of 33 cancer types. Oncomine (https://www.oncomine.org/resource/login.html) is a web-based data mining platform that assembles 86,733 samples and 715 gene expression datasets together [16]. In this study, it was employed to detect the expression level of CCNA2 in pancancer in this study. HPA (http://www.proteinatlas.org/) database was used to evaluate differences in CCNA2 expression at the protein level in pancancer. cBioPortal database (http://www.cbioportal.org) was applied to investigate the copy number alteration and mutation landscape of CCNA2 in pancancer. Patients were excluded if they (1) did not have prognostic information and (2) died within 30 days.

### 2.2. Differential Expression of CCNA2 in TCGA and SMMU Cohorts

Analysis of CCNA2 expression in multiple cancers was performed in Oncomine and TCGA databases, using *p* value < 0.05 and absolute fold change > 1.5 as the threshold. The above obtained results were validated by quantitative real-time PCR (RT-qPCR) and immunohistochemical (IHC) staining to compare differences in mRNA and protein expressions. Informed consent about the tissue sample use was obtained from each patient before initiation of the research, and the study protocol was approved by the Institutional Review Board of the Second Military Medical University (Shanghai, China) Cancer Center. Altogether, 32 paired tumor and normal tissues were collected to perform a validation experiment. The primer sequences of CCNA2 are as follows: CGCTGGCGGTACTGAAGTC (forward primer) and GAGGAACGGTGACATGCTCAT (reverse primer). Antibodies for detecting CCNA2 expression were purchased from Abcam Company (CCNA2: ab181591).

### 2.3. Prognosis Analysis of CCNA2 in TCGA Cohort

The survival information of pancancer including overall survival (OS), progression-free interval (PFI), disease-free interval (DFI), and disease-free survival (DSS) was downloaded from TCGA database for evaluating the prognostic significance of CCNA2. Additionally, ccRCC patients were divided into CCNA2-high and CCNA2-low groups based on the median expression level of CCNA2. R packages “survival” and “survminer” were used, and the Cox analysis was applied to analyze the relationship between the expression of CCNA2 and patient prognosis.

### 2.4. Enrichment Analysis of CCNA2

Based on the guilt of association of a single gene in tumors, Pearson's correlation of expression between CCNA2 and other mRNAs retrieved from TCGA transcriptome data was analyzed. Sorted by the level of the association index between genes and CCNA2, those genes most related to CCNA2 expression were selected for enrichment analysis. R package “clusterProfiler” was used to perform Gene Ontology (GO) analysis, Kyoto Encyclopedia of Genes and Genomes (KEGG) analysis, and Gene Set Enrichment Analysis (GSEA) [17].

### 2.5. Assessment of Clinical Significance of CCNA2 Expression

Clinical characteristics including the tumor stage and drug sensitivity were introduced, and the relationship between CCNA2 expression and clinical characteristics was analyzed. The datasets including IC50 (half maximal inhibitory concentration) and gene expression of cancer cell lines were downloaded from CellMiner database (https://discover.nci.nih.gov/cellminer/home.do) and GDSC (https://www.cancerrxgene.org/) database [18].

### 2.6. Differences in the Tumor Microenvironment (TME) and Therapy Response

R package “ESTIMATE” was introduced to evaluate the relationship between the degree of immune and stromal cell infiltration and the expression of CCNA2 in pancancer. Coexpression analysis of immune-related genes and CCNA2 was performed via R package “ggpubr” and “ggcor.” R package “CIBERSORT” was used to quantify the immune cell infiltration scores in pancancer, and then the correlation of the degree of immune cell infiltration and CCNA2 expression was calculated [19]. In addition, correlations between the neoantigen count, TMB, MSI and expression of T cell exhaustion marker genes, DNA mismatch repair (MMR) system genes (including MLH1, MSH2, MSH6, PMS2, and EPCAM), DNA methyltransferase (including DNMT1, DNMT2, DNMT3A, and DNMT3), ESTIMATE scores, and CCNA2 expression were analyzed. The immune infiltration scores were also calculated using the ssGSEA algorithm, and the correlation and difference between the immune cell infiltration and CCNA2 expression in ccRCC were analyzed. The impact of CCNA2 mutation on immune cell infiltration in ccRCC was analyzed by TIMER website (http://timer.cistrome.org/) [20]. CellMiner database (https://discover.nci.nih.gov/cellminer/home.do) and CCLE database (https://portals.broadinstitute.org/ccle) were implied to investigate the role of CCNA2 expression in therapy response [21, 22].

### 2.7. Statistical Analysis

Differences in CCNA2 expression in the public datasets were compared by one-way ANOVA, and differences in clinical information and response to immune checkpoint inhibitors between the two different subgroups were compared by the chi-square test. Differences in OS and PFI between the CCNA2 high-expression and low-expression groups in ccRCC patients were compared by the Kaplan-Meier method and log-rank rest. The hazard ratios (HRs) were calculated by the univariate Cox regression and multiple Cox regression analysis. All *p* values were two-sided, with *p* < 0.05 as statistically significant. Adjusted *P* value was obtained by the Benjamini-Hochberg (BH) multiple test correction. All data processing, statistical analysis, and plotting were conducted using R 4.0.4 software.

## 3. Results

### 3.1. CCNA2 mRNA Is Widely Upregulated in Cancers

**Figure 1:**
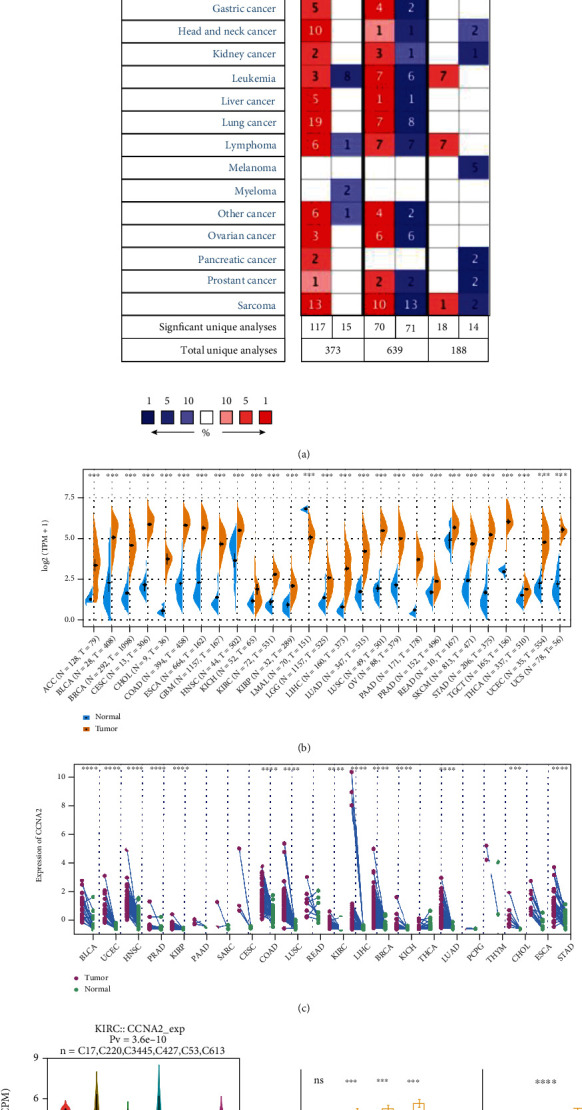
CCNA2 expression level in pancancer. (a) CCNA2 was increased in tumor tissues vs. normal tissues in Oncomine database. (b) Comparison of CCNA2 expression between tumor and normal tissues in TCGA database, ^∗^*p* < 0.05, ^∗∗^*p* < 0.01, ^∗∗∗^*p* < 0.001, and ^∗∗∗∗^*p* < 0.0001. (c) The expression level of CCNA2 was different in paired tumor and normal tissues of 22 cancer types from TCGA database, ^∗^*p* < 0.05, ^∗∗^*p* < 0.01, ^∗∗∗^*p* < 0.001, and ^∗∗∗∗^*p* < 0.0001. (d) CCNA2 expression was different in 6 ccRCC immune types: C1 (wound healing), C2 (IFN-gamma dominant), C3 (inflammatory), C4 (lymphocyte depleted), C5 (immunologically quiet), and C6 (TGF-b dominant)). (e) RT-PCR results of CCNA2 expression in the samples from SMMU cohort, *n* = 32.

The expression level of *CCNA2* in pancancer and normal tissues was analyzed firstly in Oncomine database. As shown in Figure 1(a), CCNA2 was differently expressed in most tumor and normal tissues. We next examined the differential expression of CCNA2 in TCGA database. Compared with the mRNA level in normal tissues, CCNA2 mRNA level was increased prominently in adrenocortical carcinoma (ACC), bladder urothelial carcinoma (BLCA), breast invasive carcinoma (BRCA), cervical squamous cell carcinoma and endocervical adenocarcinoma (CESC), cholangiocarcinoma (CAC), colon adenocarcinoma (COAD), esophageal carcinoma (ESCA), glioblastoma multiforme (GBM), head and neck squamous cell carcinoma (HNSC), kidney chromophobe (KICH), kidney renal clear cell carcinoma (KIRC), kidney renal papillary cell carcinoma (KIRP), brain lower grade glioma (LGG), liver hepatocellular carcinoma (LIHC), lung adenocarcinoma (LUAD), lung squamous cell carcinoma (LUSC), ovarian serous cystadenocarcinoma (OV), pancreatic adenocarcinoma (PAAD), prostate adenocarcinoma (PRAD), rectum adenocarcinoma (READ), skin cutaneous melanoma (SKCM), stomach adenocarcinoma (STAD), testicular germ cell tumor (TGCT), thyroid carcinoma (THCA), uterine corpus endometrial carcinoma (UCEC), and uterine carcinosarcoma (UCS), while it was decreased in acute myeloid leukemia (LAML) (Figure 1(b)). To make the result more creditable, the paired tumor and normal tissues in TCGA were further analyzed. As illustrated in Figure 1(c), CCNA2 was widely overexpressed in tumor tissues. In addition, the expression level of CCNA2 was significantly different in the five classic immune subtypes of ccRCC, of which C5 (immunologically quiet) was the lowest subgroup (Figure 1(d)). Through ccRCC and normal tissue from Changzheng Hospital, CCNA2 was highly expressed in tumor compared to normal tissues, which validated the above results (Figure 1(e)). To assess whether CCNA2 was expressed at different levels in various cancer stages, different pathological stages (I, II, III, and IV) of pancancer were collected. The results showed that the expression level of CCNA2 was significantly different in different stages of ACC, BRCA, COAD, KICH, KIRC, KIRP, LUAD, SKCM, TGCT, and THCA (Figure S1), suggesting that CCNA2 may play an important role in progression of various carcinomas.

### 3.2. Validation of Different Expression Levels of CCNA2 Protein

**Figure 2:**
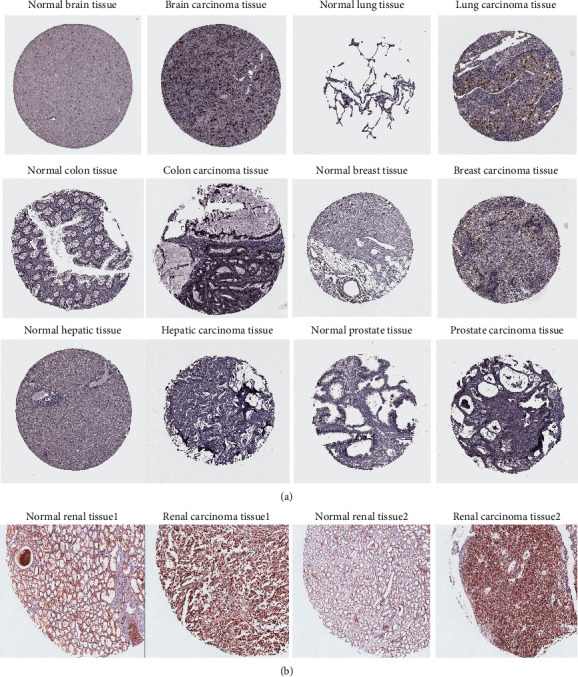
CCNA2 protein is highly expressed in tumor tissues vs. normal tissues. (a) Representative IHC images of CCNA2 expression retrieved from HPA database. (b) Representative immunohistochemical images of CCNA2 expression in ccRCC and adjacent tissues, scale bar = 50*μ*m.

To assess CCNA2 protein expression, the IHC results from the HPA database and inhouse cohort were analyzed and compared. As shown in Figure 2(a), the protein expression levels of CCNA2 were highly consistent with mRNA expression above. IHC staining showed that the expression levels of CCNA2 mRNA and protein were low in the normal brain, lung, colon, breast, liver, and prostate but high in tumor tissues. These expression differences were also validated by the paired renal tissues from SMMU cohort indicating that the expression level of CCNA2 protein in ccRCC tissues was higher than normal tissues (Figure 2(b)).

### 3.3. The Landscape of CCNA2 Mutation Profile in Various Cancers

**Figure 3:**
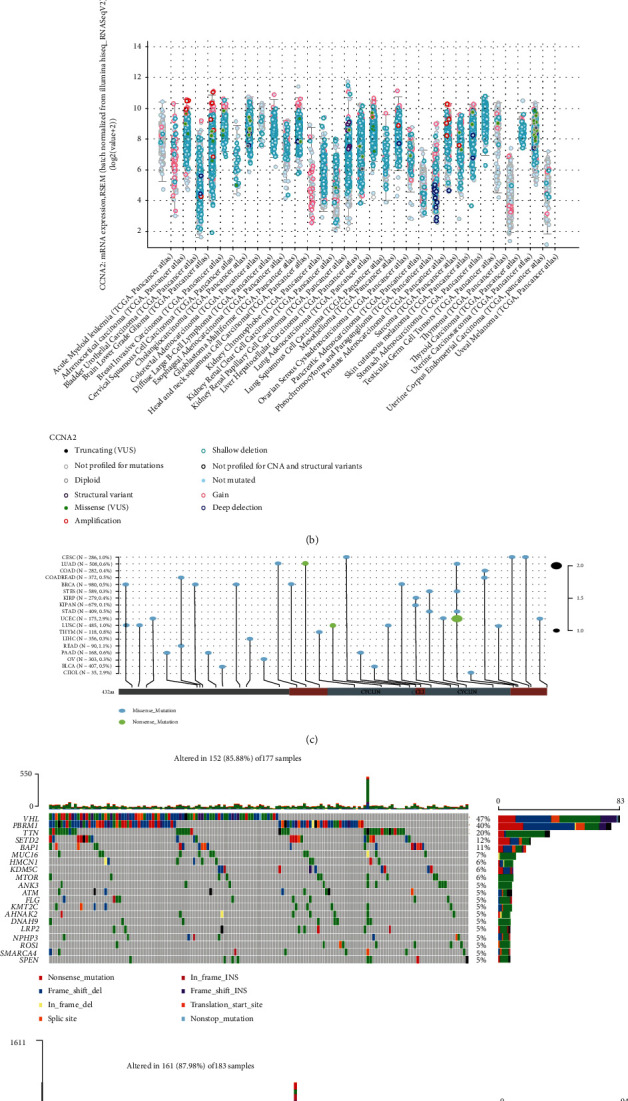
CCNA2 mutation landscape. (a and b) The mutation frequency and mutation count of CCNA2 in pancancer by cBioPortal database. (c) Mutation diagram of CCNA2 in different cancer types across protein domains. (d) The different mutation landscape in CCNA2 high- and low-expression groups in ccRCC.

The genetic alteration and DNA methylation status of CCNA2 in different tumor samples of TCGA cohorts were observed, and the result is shown in Figures 3(a) and 3(b). As shown in Figure 3(a), the highest alteration frequency of CCNA2 (>3%) appeared in UCEC patients with “mutations” as the primary type. In addition, all PRAD cases with genetic alteration (~2.5% frequency) had copy number deletion of CCNA2 (Figure 3(b)). We further analyzed the type, site, and case number of the CCNA2 genetic alteration. As shown Figure 3(c), missense mutation of CCNA2 was the main type of genetic alteration. Then, we analyzed the mutation difference between CCNA2-high and CCNA2-low subgroups in ccRCC and found that the mutation rate of PBRM1 in CCNA2-high subgroup was higher than that in CCNA2-low subgroup. These two subgroups contained different mutation profiles: MTOR, ANK3, ATM, FLG, KMT2C, AHNAK2, NPHP3, ROS1, SMARCA4, and SPEN in CCNA2-high subgroup vs. DNAH9, ARID1A, DST, PKHD1, SYNE1, ATRX, CSDM3, MACF1, MUC4, and SPTA1 in CCNA2-low subgroup (Figure 3(d)).

### 3.4. Correlation of TREM2 Expression with DNA Methylation and RNA Modification

**Figure 4:**
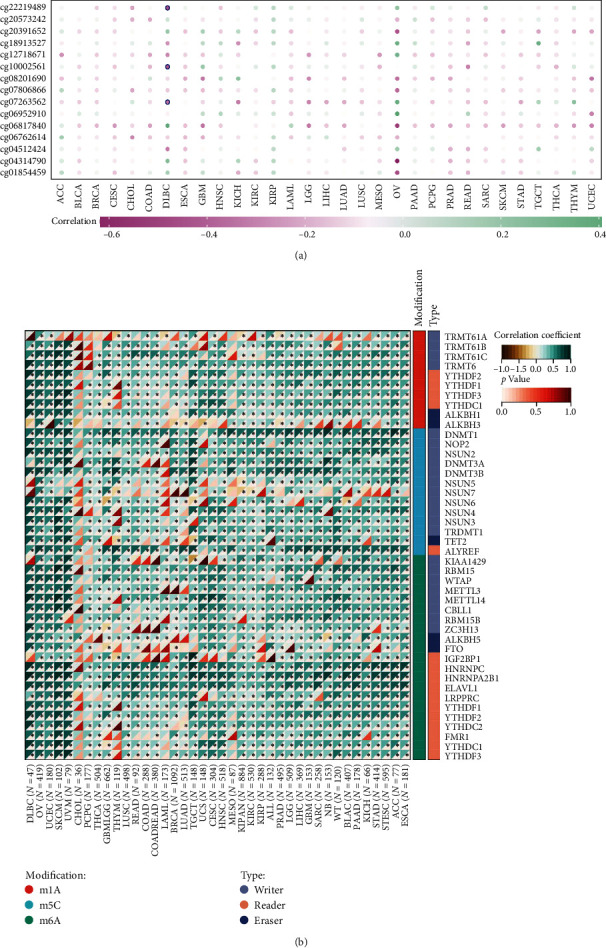
DNA methylation and RNA modification in CCNA2. (a) The correlation of CCNA2 expression and methylation degree in pancancer. (c) The correlation of CCNA2 expression and RNA modification regulator expression in pancancer.

To further analyze the potential regulation effect of DNA methylation and RNA modification in CCNA2 expression, firstly, we systematically explored the correlation of DNA methylation level and CCNA2 expression, which indicated that DNA methylation could negatively regulated CCNA2 expression in BLCA via cg22219489, cg10002561, and cg07263562(Figure 4(a)); and RNA modification-related genes (including m1A, m5C, and m6A) were also significantly positive correlated with CCNA2 expression (Figure 4(b)). All those results indicated that CCNA2 expression might mainly regulated via RNA posttranscriptional modification.

### 3.5. The Association between CCNA2 mRNA Expression and Clinical Outcomes in Cancers

**Figure 5:**
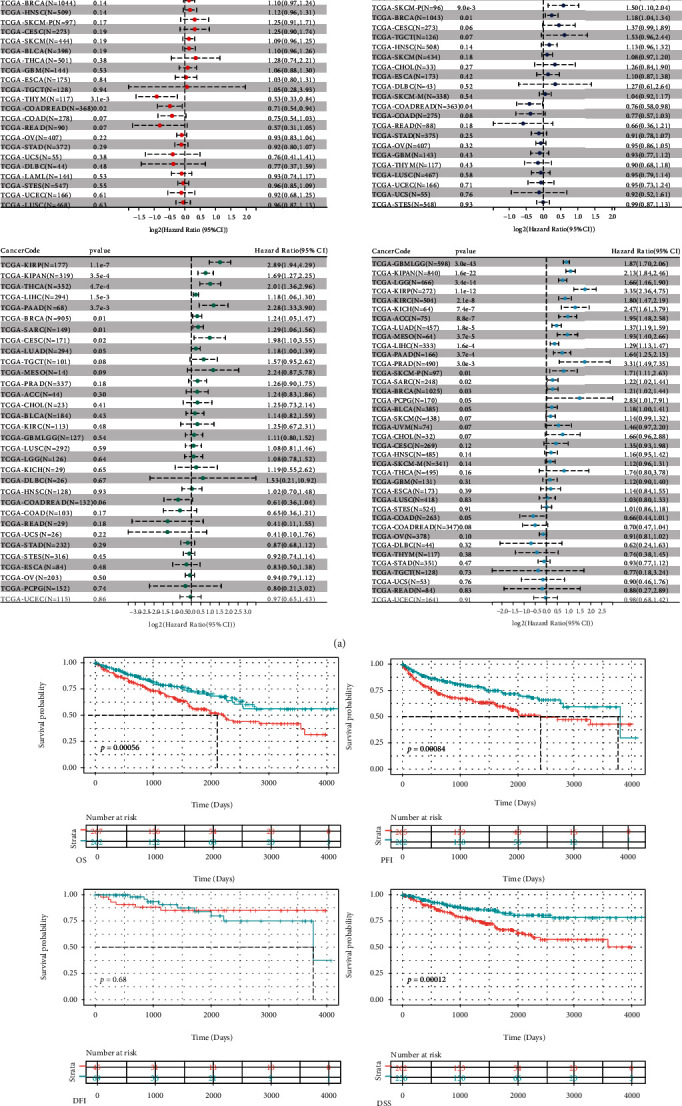
The prognostic significance of CCNA2 in various cancer types. (a) The impact of CCNA2 on OS, PFI, DFI, and DSS in pancancer using the Cox proportional hazard models. (b) The impact of CCNA2 on OS, PFI, DFI, and DSS in ccRCC using the Kaplan-Meier analysis.

The association of CCNA2 expression with OS, PFI, DFI, and DSS in 33 cancer types in TCGA is shown in Figure 5(a), demonstrating a significant relationship between CCNA2 expression and prognosis in ACC, COAD, KICH, KIRC, KIRP, LGG, LIHC, LUAD, MESO, PAAD, PRAD, SARC, THYM, and UVM. Among them, the expression of CCNA2 was most significantly associated with the prognosis of KIRC. As shown in Figure 5(b), KIRC patients with high CCNA2 expression had a worse prognosis.

### 3.6. The Correlation of CCNA2 Expression and Canonical Tumorous Hallmarks

**Figure 6:**
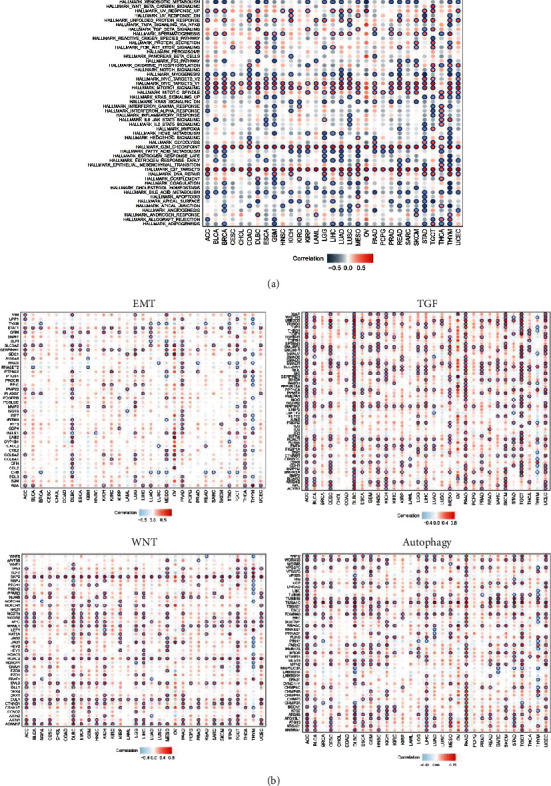
Association between CCNA2 and hallmarks in pancancer. (a) Correlations of CCNA2 expression with HALLMARKS enrichment score in 33 cancer types. (b) Correlation between CCNA2 expression and EMT, TGF, WNT, and autophagy pathway in pancancer.

To explore the biological function of CCNA2 in different cancer types, we firstly utilized the GSVA to calculate the enrichment scores of 50 canonical tumor associated pathways in pancancer level; then, the correlation of those enrichment scores and CCNA2 expression was estimated. The results indicated that CCNA2 mainly positively regulated Myc, G2M checkpoint, and E2F_target pathways while negatively regulated xenobiotic metabolism, myogenesis, fatty acid metabolism, and bile acid metabolism in most cancer types (Figure 6(a)). In addition, CCNA2 participate nearly all the canonical hallmarks in THYM, which indicated that CCNA2 hold a leading role in the occurrence and development of THYM and could be treated as a promising treatment target. We also explored other aetiological pathways determining the clinical outcomes, including EMT, TGF, WNT, and autophagy pathways (Figure 6(b)). Interestingly, CCNA2 could activate those signals in most cancer types. CCNA2 might significantly activate EMT pathways in ACC, DLBC, LGG, MESO, PAAD, and THCA while inhibit EMT in THYM. Among TGF signal, CCNA2 could activate such a pathway in ACC, BLCA, CESC, DLBC, HNSC, KICH, LGG, LIHC, MESO, PAAD, SKCM, TGCT, and UCEC. In WNT signal, CCNA2 also stimulate this pathway in ACC, BLCA, CESC, DLBC, ESCA, KICH, LGG, LIHC, MESO, PAAD, TGCT, and UCEC. And CCNA2 might also participate in autophagy pathway especially in ACC, BLCA, CESC, DLBC, ESCA, GBM, HNSC, KICH, LIHC, MESO, PAAD, READ, TGCT, and UCEC.

### 3.7. The Biological Function of CCNA2 in ccRCC

**Figure 7:**
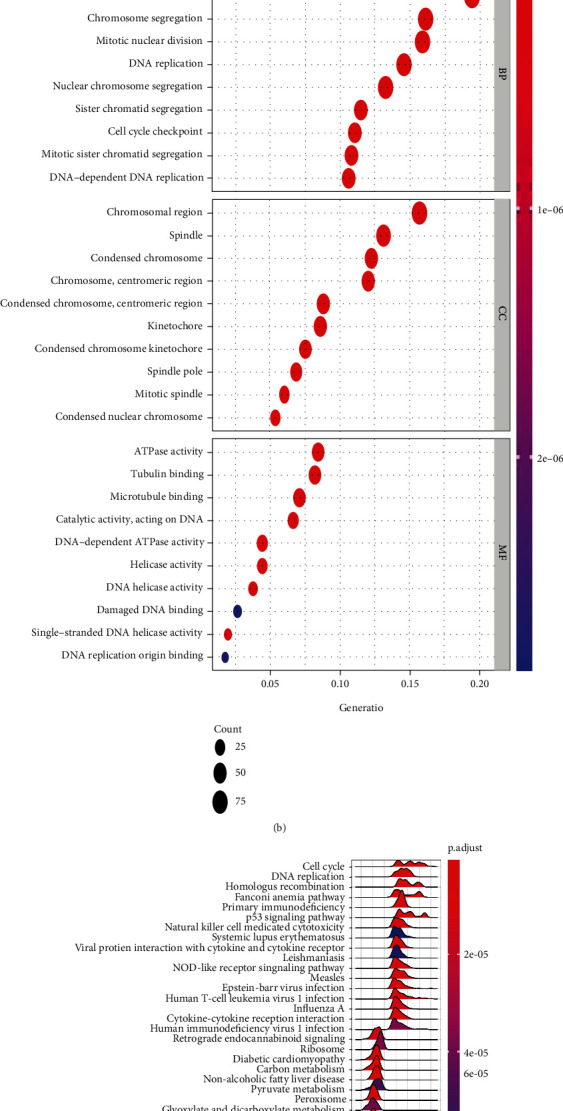
The biological function of CCNA2 in ccRCC. (a) Heatmap of association between estimated scores and DDX39 in TCGA ccRCC patients. (b–d) GO, KEGG, and GSEA analysis of CCNA2 in ccRCC.

Based on the character of “guilt of association” in the controlling networks in tumor, we use the transcriptome data collected from TCGA database, and the top 500 mRNAs mostly related to CCNA2 were retrieved via Spearman's correlation analysis. The correlation of those genes was presented in Figure 7(a). The enrichment function was performed based on those significantly related genes. GO analysis indicated that biological process module mainly enriched in organelle fission, nuclear division, chromosome segregation, mitotic nuclear division, and nuclear chromosome segregation; and the cellular component mainly involved in chromosomal region, spindle, and condensed chromosome; and molecular function centered on ATPase activity, tubulin binding, and microtubule binding (Figure 7(b)). KEGG results indicated that series of pathways related to CCNA2 in ccRCC, including cell cycle, DNA replication, homologous recombination, primary immunodeficiency, p53 signaling pathways, and natural killer cell-mediated cytotoxicity pathways (Figure 7(c)). GSEA further validated that CCNA2 could activate E2F targets, interferon alpha response, IL6-JAK-STAT signaling, interferon gamma response, Myc targets, G2M checkpoint, and inflammatory response while inhibit fatty acid metabolism and bile acid metabolism pathways in ccRCC (Figure 7(d)).

### 3.8. CCNA2 Is Associated with Tumor Evasion via Different Mechanisms by Infiltration Immune Cells

**Figure 8:**
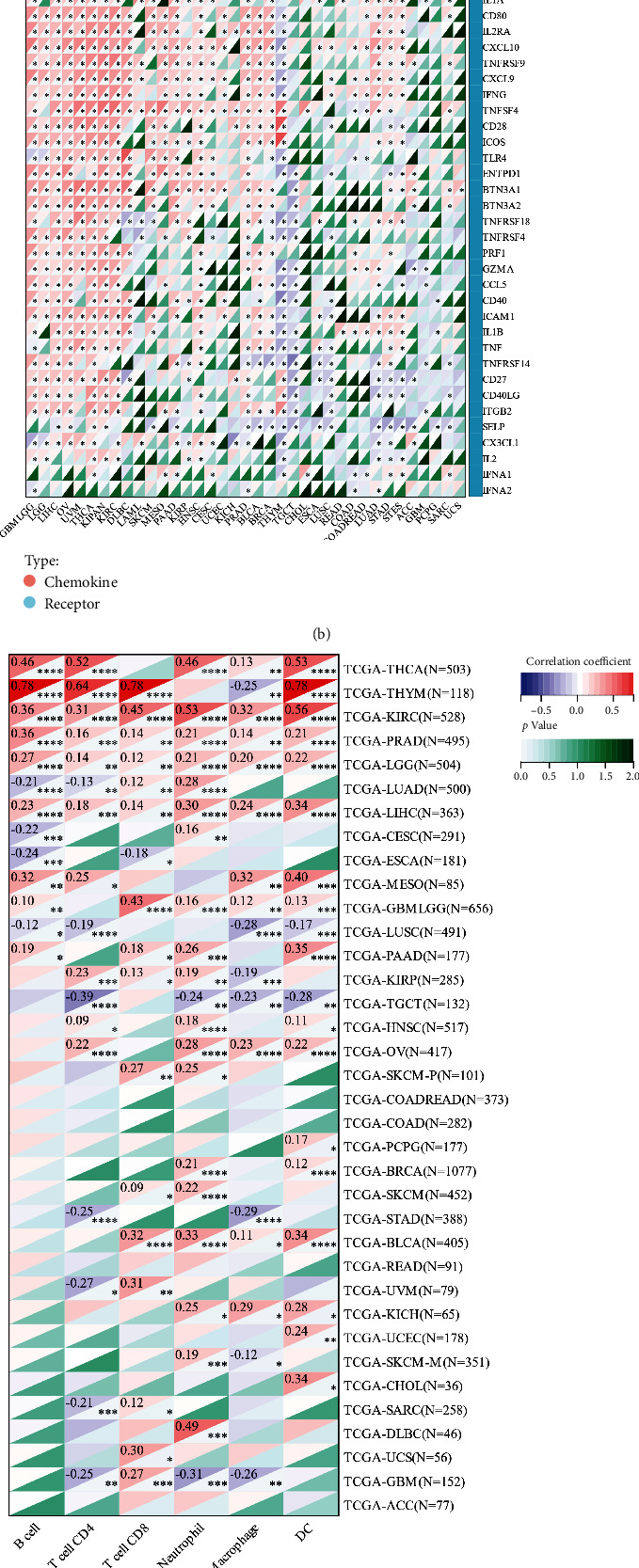
Association between CCNA2 and tumor immunity in pancancer. (a and b) Correlations of CCNA2 expression with immune moderator genes and immune checkpoint-related genes in 33 cancer types. (c, d, and e) Correlation between CCNA2 expression and immune cell infiltration via TIMER, EPIC, and CIBERSORT algorithms.

To explore the immune function of CCNA2 in pancancer types, the correlation between immune-regulated genes, immune checkpoint inhibitor genes, and CCNA2 expression was calculated firstly (Figures 8(a) and 8(b)). CCNA2 could positively regulate chemokine, chemokine receptors, MHC, immunoinhibitor, and immunostimulator in UVM, OV, THCA, KIPAN, KIRC, PAAD, GBM, LGG, DLBC, LIHC, PARD, BLCA, MESO, KIRP, and KICH while negatively regulate in THYM and TGCT. In addition, CCNA2 could positively regulate TAP1, TAP2, CD276, MICB, PVR, and ULBP1 in almost all the cancer types. We also found that CCNA2 could significantly regulate most immunomodulators in various cancer types, which indicated that CCNA2 could determine immunotherapy benefit of those cancer types. To detect whether CCNA2 could influence the process of immune cell infiltration degree in pancancer, we utilized three algorithms to evaluate the immune infiltration degree and calculate the correlation index between CCNA2 expression and immune cell infiltration degree. As Figure 8(c) indicated, CCNA2 was most significantly related with B cell, CD4 T cell, CD8 T cell, and DC cell in THCA, THYM, and KIRC. In addition, CCNA2 was also significantly correlated with endothelial cell and macrophage cell in various cancers, especially in THYM (Figure 8(d)). Finally, CIBERSORT was employed to evaluate the immune cell infiltration degree in pancancer, which illustrated that CCNA2 could determine M1 macrophage in nearly all the cancer type, which further validated the results above (Figure 8(e)). All those results indicated that CCNA2 could regulate immune cell infiltration via different mechanism under various tumor microenvironments, and further experiment needs to decipher the heterogeneous mechanisms.

### 3.9. Correlation of CCNA2 Expression and Stemness, TMB, MSI, MMR, and DNA Methyltransferases

**Figure 9:**
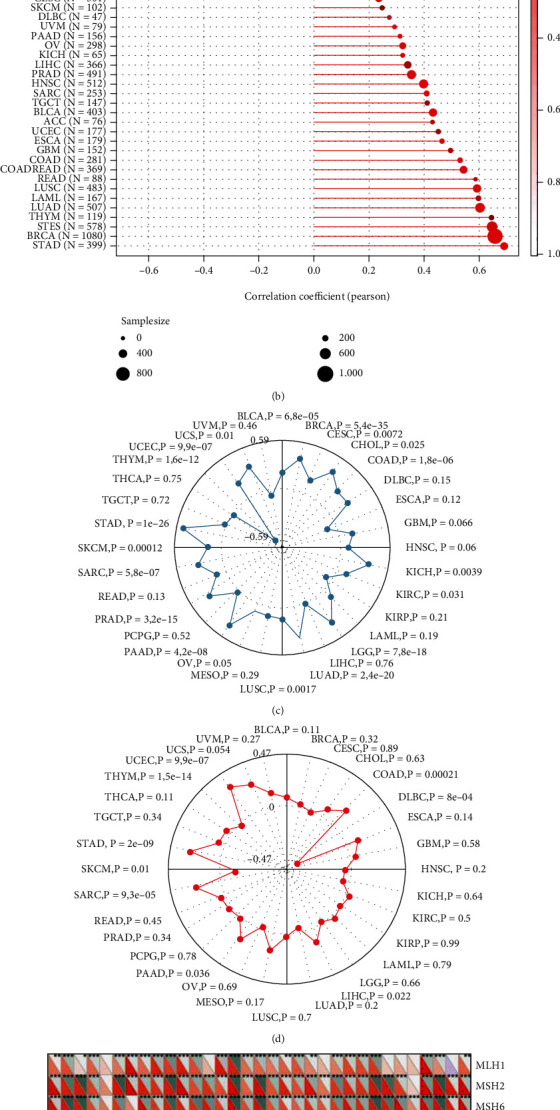
Correlations of CCNA2 with Stemness index, TMB, MSI, MMR, and DNA methyltransferases in pancancer. (a and b) Correlations of CCNA2 expression with DNAss and RNAss index in 33 cancer types. (c) Correlations of CCNA2 expression with TMB in 33 cancer types. (d) Correlations of CCNA2 expression with MSI in 33 cancer types. (e) Correlations of CCNA2 expression with expression levels of five MMR genes in 33 cancer types. (f) Correlations of CCNA2 expression with four DNA methyltransferases in 33 cancer types.

Since tumor is composed of heterogeneous cell clusters holding various degrees of functional and genetic heterogeneity, cancer stem cells (CSCs) are capable to maintain tumor survival via genetic and epigenetic factors, accelerate tumor metastasis, resist drug, and maintain tumor microenvironment [23]. Herein, we explored the association of CCNA2 expression and stemness including DNAss and RNAss. CCNA2 expression was significantly positively correlated with DNAss in GBM (*r* > 0.5) while negatively correlated in THYM (*r* < −0.6). In addition, CCNA2 expression was positively related to STAD, BRCA, STES, and THYM in RNAss (*r* > 0.6) (Figures 9(a) and 9(b)). TMB has been extensively studied and is suggested to play a vital role in tumor-responsiveness to immune checkpoint blockade [24]. MSI refers to the change in microsatellite sequence length caused by insertion or deletion mutation during DNA replication, which is often caused by MMR defects [25]. The relationship between CCNA2 expression and TMB and MSI in pancancer was also analyzed and displayed as a radar chart. As shown in Figures 9(c) and 9(d), CCNA2 high expression was positively correlated with TMB in BLCA, BRCA, CESC, CHOL, COAD, KICH, KIRC, LGG, LUAD, LUSC, PAAD, PRAD, SARC, SKCM, STAD, UCEC, and UCS and negatively correlated with THYM. CCNA2 high expression was also positively correlated with MSI in COAD, LIHC, PAAD, SARC, STAD, and UCEC but negatively correlated with MSI in DLBC and SKCM. MMR is a repair method that restores the normal nucleotide sequence in DNA molecules containing mismatched bases [26]. It is mainly used to correct mismatched base pairs on DNA double helix. The correlation between CCNA2 expression and MLH1, MSH2, MSH6, PMS2, and EPCAM is illuminated in Figure 9(e), showing that CCNA2 expression was positively correlated with MLH1 in 26 cancer types, with MSH2 and MSH6 in 31 cancer types, with PMS2 in 24 cancer types, and with EPCAM in 15 cancer types. On the contrary, there was a negative correlation between CCNA2 expression and EPCAM in LGG and THYM. DNA methylation is one of the important mechanisms of gene epigenetics, which is involved in the process of regulating gene expression and cell differentiation [27]. The relationship between four methyltransferases was analyzed, and the results showed that the expression level of CCNA2 was positively correlated with all four methyltransferases in 23 cancer types (Figure 9(f)). Meanwhile, it was not correlated with any of the four methyltransferases in UCS and CHOL or with DNMT2 in LUAD, PAAD, and THCA, DNMT3A in DLBC, LAML, PAAD, and READ, and DNMT3B in LAML and PAAD.

### 3.10. Relationship between CCNA2 and Tumor Immune Microenvironment in ccRCC

**Figure 10:**
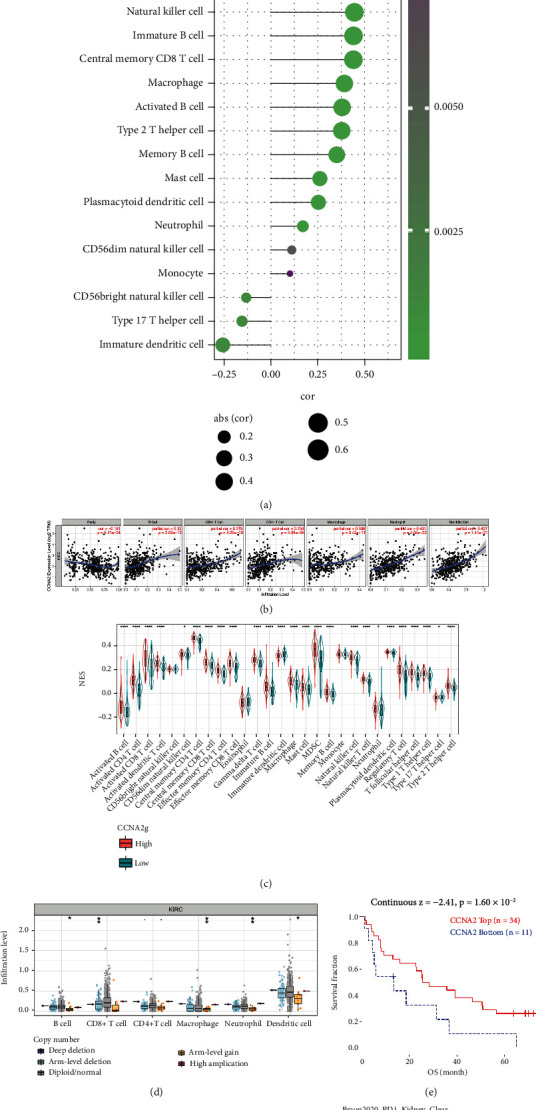
CCNA2 expression and mutation are related with immune infiltration in ccRCC. (a) Association between CCNA2 and NES of immune cells in ccRCC. (b) CCNA2 is correlated with multiple immune cell infiltration in TIMER database. (c) The different immune infiltration degrees in CCNA2 high- and CCNA2 low-expression groups in ccRCC. (d) Impact of CCNA2 mutation on immune cell infiltration. (e and f) Kaplan-Meier curves of survival ratios, OS, and PFS, as a measure of the immunotherapeutic response between ccRCC cohort with high- and low-expression level of CCNA2. (g) Difference of prognostic value including CCNA2 and other biomarkers or indexes. ∗ indicates *p* < 0.05, ∗∗ indicates *p* < 0.01, and ∗∗∗ indicates *p* < 0.001.

Renal cell carcinoma (RCC) is one of the earliest tumor types to use immunotherapy. As early as in the 1980s, interferon was used for advanced RCC [28]. With the emergence of immune checkpoint inhibitors, immunotherapy has ushered in a new era in the treatment field of RCC [29]. In view of the above positive results of CCNA2 analysis in KIRC, we comprehensively analyzed the relationship between CCNA2 expression and KIRC immune cell infiltration using multiple databases and found that CCNA2 expression was positively correlated with the infiltration of various immune cells, especially with T cell cells, which includes activated CD4 T cell, regulatory T cell, central memory CD4 T cell, effector memory CD4 T cell, type 1 T helper cell, MDSC, and T follicular he (Figure 10(a)). TIMER dataset indicated that CCNA2 expression was negatively correlated with tumor purity, thus enhancing infiltration of several immune cell types in ccRCC, including B cell, CD8^+^ T cell, CD4+ T cell, macrophage, neutrophil, and dendritic cell (Figure 10(b)). ssGSEA analysis also identified that multiple immune cell types infiltrated differently between CCNA2-high and CCNA2-low subgroups (Figure 10(c)).  Figure 10(d) shows the effect of CCNA2 mutation on the degree of immune cell infiltration. There were significant differences in the degree of cell infiltration between diploid/normal and arm-level gain in B cell, macrophage, neutrophil, and dendritic cell and between arm-level deletion and diploid/normal in CD8+ T cell. In addition, CCNA2 was a protective factor for OS and PFS in ccRCC immune checkpoint inhibitor cohort (Figures 10(e) and 10(f)); CCNA2 performed better in immune response rate than any other signature (including TIDE, MSI score, mutation, CD274, CD8, IFNG, T-Clonality, B-Clonality, and Merck18) in ccRCC cohort (Figure 10(g)).

### 3.11. Correlation Analysis between CCNA2 Expression and Drug Sensitivity

**Figure 11:**
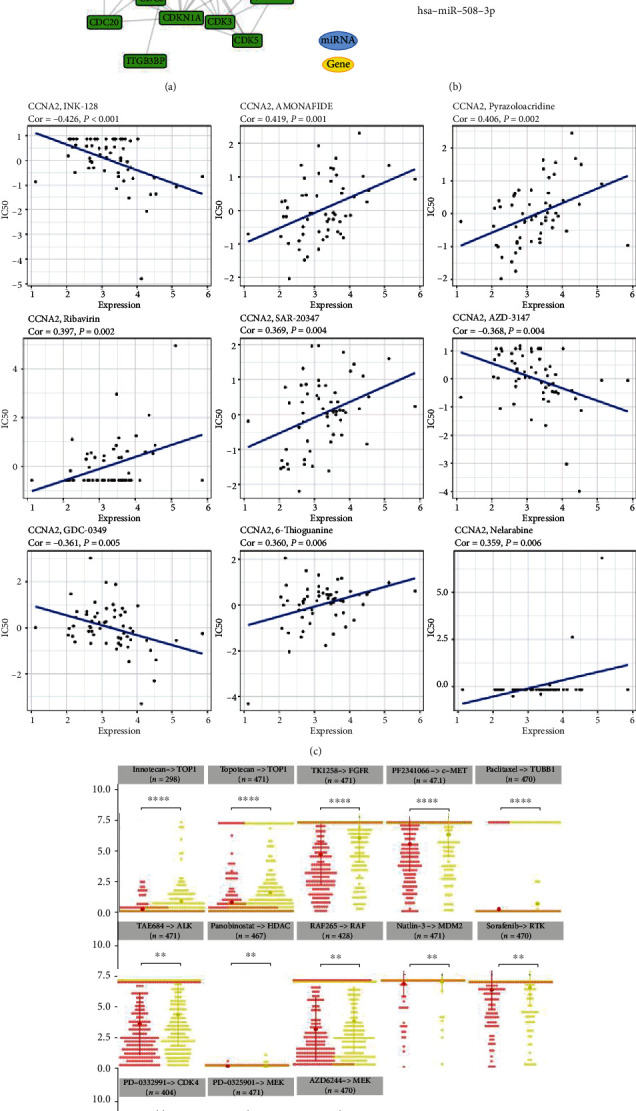
Correlation analysis between CCNA2 expression and drug sensitivity. (a) Network of functional gene partners of CCNA2 and mRNAs. (b) The ceRNA network of CCNA2 in cancers. (c) Correlation between CCNA2 and sensitivity of the top 9 anticancer drugs in CellMiner database. (d) Difference of drug sensitivity between CCNA2 high- and low-expression groups in GDSC database.

To decipher the functional partners of CCNA2 in cancers, gene network interaction analysis was performed in Figures 11(a) and 11(b), and the most relevant mRNA and miRNA were detected. As shown in Figure 11(a), CCNA2 expression was positively correlated with resistance of INK-128, AZD-3147, and GDC-0349, as well as with sensitivity of amonafide, pyrazoloacridine, ribavirin, SAR-20347, 6-thioguanine, and nelarabine. According to GDSC database, we found that high expression of CCNA2 could make cancer more sensitive to irinotecan (target at TOP1), topetecan (target at TOP1), TKI258 (target at FGFR), PF2341066 (target at c-MET), paclitaxel (target at TUBB1), TAE684 (target at ALK), panobinostat (target at HDAC), RAF265 (target at RAF), nutlin-3 (target at MDM2), sorafenib (target at RTK), and PD-032991 (target at CDK4) and resistant to AZD6244 (target at MEK) (Figure 11(b)).

## 4. Discussion

Our results indicated that CCNA2 is significantly related to the occurrence and progresses of various cancer types. Prior studies also reported functional relation between CCNA2 and clinical diseases, especially tumors [30]. Whether CCNA2 involves in the tumor microenvironment and pathogenesis of different tumors through common or specific mechanisms remains unclear. Our study illustrated the correlation of expression level and genomic alternation of CCNA2 with tumor staging, progression, tumor immunity, and drug sensitivity across pancancer and subtypes, especially in ccRCC.

Based on the genomic data collected from a variety of cancer types and a profound understanding about the biology and pathology of pancancer, TCGA has developed a series of therapeutic strategies for the treatment of various cancer types [14]. CCNA2 is a cyclin present in mammals and promotes S-phase progression and G2-M phase transition by binding CDK in the mitotic cell cycle [31]. CCNA2 is located on chromosome 4 and is encoded by human CCNA2, belonging to the highly conserved cyclin family. It is reported to be correlated with cytoskeleton dynamics and cell motility [32]. It was found in our study that CCNA2 was involved in tumor proliferation, invasion, and differentiation, thus could be treated as a novel and promising diagnostic also therapeutic targets for cancers.

In this study, we first disclosed that the mRNA expression of CCNA2 was upregulated in ACC, BLCA, BRCA, CESC, CHOL, COAD, ESCA, GBM, HNSC, KICH, KIRC, KRIP, LGG, LIHC, LUAD, LUSC, OV, PAAD, PRAD, READ, SKCM, STAD, TGCT, THCA, UCEC, and UCS cancer tissues vs. normal tissues by using TCGA, which indicated that CCNA2 might be an oncogenic molecule in tumorigenesis. Based on the IHC results from HPA database and SMMU cohort, we validate that CCNA2 expression was more pronounced in the corresponding tumor tissues vs. normal kidney, intestine, liver, lung, breast, prostate, and brain tissues. Furthermore, based on survival curves, we demonstrated CCNA2 mRNA expression as a dependable diagnostic factor, implying that CCNA2 is a possibly promising biomarker for pancancer diagnosis. More importantly, CCNA2 mRNA expression was significantly correlated with the patient prognosis in ACC, COAD, KICH, KIRC, KIRP, LGG, LIHC, LUAD, MESO, PAAD, PRAD, SARC, THYM, and UVM, suggesting that CCNA2 plays an important role in the progression of pancancer, especially KIRC patients with high expression of CCNA2 holding a worse prognosis. Our results are consistent with previous studies and clinical trials, which concluded that CCNA2 was highly expressed in multiple cancers and patients with high CCNA2 expression owning high-risk features. Gao et al. reported that CCNA2 is highly expressed in breast cancer and could be treated as a power predicative marker in BLCA patients [33]. Gan et al. also found that CCNA2 is overexpressed and acts as a novel biomarker in regulating the growth and apoptosis in colorectal cancer [34].

To further decipher the biological role of CCNA2 in the cancers and ccRCC, several enrichment function algorithms were implied. KEGG pathway results were primarily enriched in oocyte meiosis, cell cycle, pyrimidine metabolism, asthma, alpha linolenic acid metabolism, arachidonic acid metabolism, and linoleic acid metabolism. Meanwhile, GSEA results suggested that CCNA2 may be correlated with coagulation, KRAS signaling, myogenesis, bile acid metabolism, mtorc1 signaling, E2F targets, and G2M checkpoint. Ruan et al. found that CCNA2 could facilitate epithelial-to-mesenchymal transition via integrin *α*v*β*3 signaling in non-small-cell lung carcinoma [35]. CCNA2 could also act as a significant downstream facilitating tumor progression. Chen et al. suggested that ROBO1 could promote the development of pancreatic cancer via CCNA2/CDK axis [36]. Interestingly, our study illustrated that CCNA2 could enhance the development of ccRCC via several immune-related signals, including immunodeficiency, natural killer cell-mediated cytotoxicity pathway, and IL6-JAK-STAT3 pathways. Further study is required to clarify the detailed mechanism underlying the role of CCNA2 in impacting those signaling pathways in cancers and ccRCC.

TMB, MSI, and neoantigen indexes are two widely studied biomarkers which may have a profound impact on the response to tumor immunotherapy and patient survival [37]. However, those indexes only performed well in several types of tumors and hold high test costs. Consequently, it is urgent to discover new and economic biomarker for predicting immune therapy response. The results of our study showed that upregulation of CCNA2 mRNA was significantly and positively correlated with TMB, MSI, and neoantigen indexes in BLCA, BRCA, CESC, CHOL, COAD, KICH, KIRC, LGG, LUAD, LUSC, PAAD, PRAD, SARC, SKCM, STAD, UCEC, and UCS. The levels of DNA methylation and MMR state in tumors have increasingly been recognized as a promising index for evaluating efficacy of target and immune therapy [26, 38]. The current study also found that CCNA2 expression was positively correlated with DNA methylation and MMR in most cancers, which suggested that CCNA2 might influence DNA methylation level or MMR state, thus determining the clinical outcomes of cancer patients [39]. Some other interesting and relevant discoveries include the significant correlation between CCNA2 expression and MLH1, MSH2, MSH6, PMS2, and EPCAM, suggesting that CCNA2 may be involved in tumor-related developmental processes including signaling, migration, and proliferation [40, 41]. Zhou et al. found that CCNA2 is a potential diagnostic and prognostic biomarker for LUAD, and CCNA2 expression positively correlated with immunity therapy efficiency in LUAD [42]. Chen et al. also confirmed that CCNA2 is involved in hypoxia signature affecting the clinical outcomes and immune microenvironment of ACC [43]. To date, only several studies assessed the immune role of CCNA2 in cancers. Our findings may shed lights on the molecular mechanisms underlying tumor progression and metastasis and provide useful clues for seeking strategies for the clinical treatment and prognostic prediction of cancers.

TME and tumor evasion are correlated with cancer prognoses and therapeutic [8]. There are two major mechanisms of immune evasion until now, which are consisted of dysfunctional T cell phenotypes and T cell exclusion [7, 44]. However, considering the complex compositions and communication of TME, it is necessary to identify the key molecules in TME [9]. Our study indicated that CCNA2 expression was significantly and positively correlated with the level of CD 4 T+ cell memory cells and M0 and M1 macrophage infiltration across various cancer types. Interestingly, CCNA2 is found significantly positively correlated with immune therapy response in BLCA, BRCA, KIRC, LGG, LUAD, PRAD, and THCA while negatively correlated with ACC, GBM, and TGCT. Wang et al. found that CCNA2 functions as a potential immune therapy maker in BLCA [45]; Xu et al. also confirmed that CCNA2 could activate macrophages, thus enhancing tumor immunity [46]. Also, CCNA2 expression was positively correlated with ImmuneScore in THCA and KIRC and negatively correlated with ImmuneScore in BRCA, LUSC, and GBM. Additionally, we found that CCNA2 was significantly correlated with immune checkpoint-related genes in KICH, KIRC, and THCA. Considering with complex and heterogeneity of TME and lack of robust immune biomarkers in KICH, KIRC, and THCA, CCNA2 seemed to be new indicator for those cancers. In a word, all these findings suggest that CCNA2 might play a significant role in TME and could regulate several key immune cell functions, though further study is required to verify these conclusions.

With the rapid development of immunotherapy for ccRCC in recent years, resistance, increasing recurrence rates, and side effects have surfaced, and there are indications that new biomarkers or therapy target for predicting efficacy and prognosis are in urgent need of discovery [47]. Although ccRCC is deemed as immune cell infiltration-high tumor, it is also notorious for immune dysfunction and depletion [48–50]. It is urgent to comprehensively analyze the mechanism of this abrogated immunity phenomenon in ccRCC. We utilized correlation analysis to determine the influence of CCNA2 expression in a series of immune cells in ccRCC. Interestingly, our results found that CCNA2 expression significantly related most of immune cell infiltration in ccRCC, among which CCNA2 positively correlated with several T cell types including activated CD4^+^ T cell, regulatory T cells, central memory CD4^+^ T cells, and effector CD4^+^ T cell while negatively correlated with immature dendritic cell and Type17 T cells. The results from TIMER database further validated those result. Datasets from Braun et al. research further proved that CCNA2 could indicate a robust prognosis in CCNA2 high-expressed group who received immune checkpoint inhibitors therapy and thus CCNA2 could work as well-performed indicator for ICB sensitivity compared with other reported makers or signatures [51]. All results remind us of that CCNA2 as a valid and reliable evaluation biomarker with the utility for easy application in ccRCC patient clinical management.

During the past decades, chemotherapy remains the mainline treatment choice for later stage patients, while drug resistance is responsible for over 80% deaths in cancer patients receiving chemotherapy or target dugs [4, 52]. Considering such phenomenon and the burden to develop new drugs, our study also systematically analyzed the correlation of CCNA2 expression and drug sensitivity to explore the potential drug target at CCNA2. We found that CCNA2 expression was positively related to amonafide, pyrazoloacridine, ribavirin, SAR-20347, and 6-thioguanine. Amonafide shows a potential therapeutic effect on breast cancer, acute myeloid leukemia, and melanoma [53–55]. Pyrazoloacridine holds significant antitumor effect on several cancer types including breast cancer, neuroblastoma, and glioma [56–58]. Hence, these candidate molecular drugs might also possess potential efficacy for CCNA2 highly expressed cancer types.

However, there are still some limitations in our study. Firstly, our study was mainly based on bioinformatics analysis, which implies that there were few experiments to confirm our statements, which need further systematic experiments to validate those results. Secondly, we only perform IHC and RT-qPCR to detect the different expression level on ccRCC patients, which is urgent to decipher the detailed role of CCNA2 in ccRCC patients.

## 5. Conclusion

To sum up, our results suggest that CCNA2 expression was upregulated in pancancer tissues including RCC vs. normal tissues. In addition, high CCNA2 expression was correlated with poor clinicopathological features in various cancers, especially for ccRCC. CCNA2 might play pivotal pathogenic roles in the immuneoncology context of the TME. The strong association of CCNA2 with tumor immunity suggests that CCNA2 may prove to be a promising therapeutic target for immunotherapy.

## Data Availability

The data used to support the findings of this study have been deposited in the TCGA repository (https://portal.gdc.cancer.gov/).

## Supplementary Materials

Supplementary Materials Association between CCNA2 expression and tumor stage in pancancer.
