# Supplementary figures and images for: CCNA2 as an Immunological Biomarker Encompassing Tumor Microenvironment and Therapeutic Response in Multiple Cancer Types
Source: Oxid Med Cell Longev. 2022 Mar 31;2022:5910575. doi: 10.1155/2022/5910575 (PMC8989596; doi:10.1155/2022/5910575)

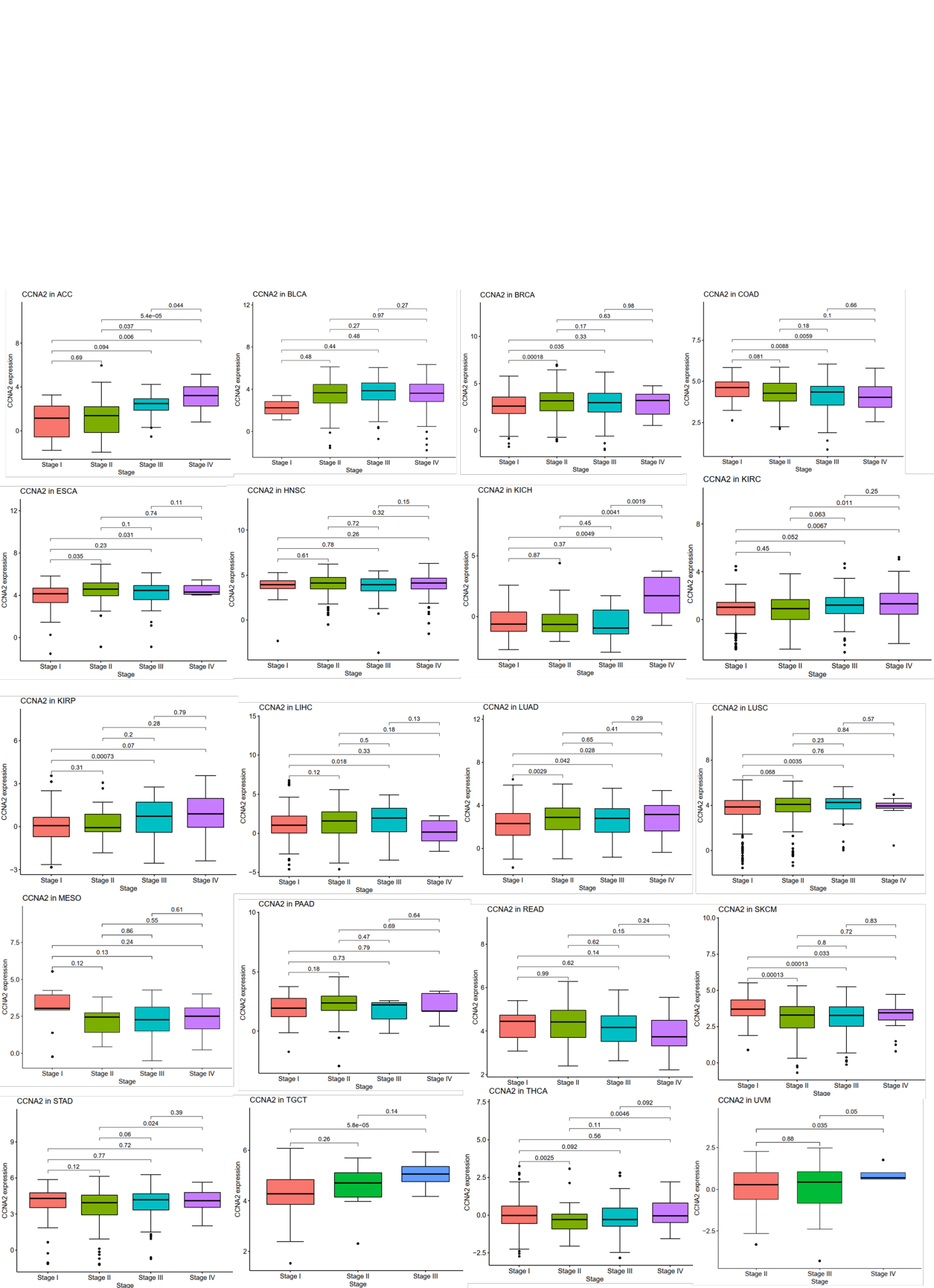

Supplement: Supplementary Materials — Association between CCNA2 expression and tumor stage in pancancer. [file 5910575.f1.pdf]
